# Bis{2-[3-(hy­droxy­imino-κ*N*)butan-2-yl­idene]-*N*-methyl­hydrazinecarbothio­amide-κ^2^ *N* ^2^,*S*}nickel(II) dichloride

**DOI:** 10.1107/S1600536811055395

**Published:** 2012-01-07

**Authors:** Halema Shaban Abduelftah, Amna Qasem Ali, Naser Eltaher Eltayeb, Siang Guan Teoh, Hoong-Kun Fun

**Affiliations:** a School of Chemical Sciences, Universiti Sains Malaysia, Minden, Penang, Malaysia; b Faculty of Science, Sabha University, Libya; c Department of Chemistry, International University of Africa, Sudan; d X-ray Crystallography Unit, School of Physics, Universiti Sains Malaysia, 11800 USM, Penang, Malaysia

## Abstract

The asymmetric unit of the title compound, [Ni(C_6_H_12_N_4_OS)_2_]Cl_2_, contains two independent Ni^II^ complex cations and four chloride anions. Each Ni^II^ ion is six-coordinated in a distorted octa­hedral geometry by four N atoms from the two imine and two oxime groups and two S atoms from the thione group. In the crystal, the cations and anions are linked through N—H⋯Cl and O—H⋯Cl hydrogen bonds into infinite chains propagating along [10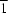]. Weak inter­molecular C—H⋯O and C—H⋯Cl hydrogen bonds are also observed.

## Related literature

For bond-length data, see: Allen *et al.* (1987 ▶). For related structures, see: Abduelftah *et al.* (2012*a* ▶,*b* ▶); Choi *et al.* (2008 ▶). For the biological activity, pharmacological properties and analytical applications of thio­semicarbazones and their metal complexes, see: Cowley *et al.* (2002 ▶); Ming (2003 ▶); Lobana *et al.* (2004 ▶, 2007 ▶).
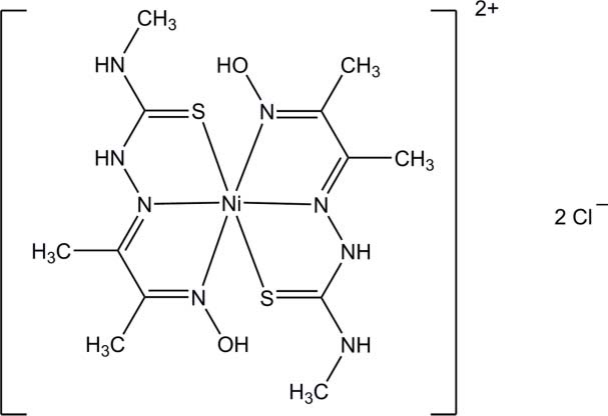


## Experimental

### 

#### Crystal data

[Ni(C_6_H_12_N_4_OS)_2_]Cl_2_
*M* *_r_* = 506.12
Monoclinic, 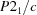

*a* = 8.9484 (1) Å
*b* = 13.8043 (2) Å
*c* = 35.4643 (5) Å
β = 95.780 (1)°
*V* = 4358.5 (1) Å^3^
*Z* = 8
Mo *K*α radiation
μ = 1.35 mm^−1^
*T* = 100 K
0.43 × 0.16 × 0.06 mm

#### Data collection

Bruker APEXII CCD diffractometer
Absorption correction: multi-scan (*SADABS*; Bruker, 2005 ▶) *T*_min_ = 0.595, *T*_max_ = 0.925
53587 measured reflections
13546 independent reflections
9309 reflections with *I* > 2σ(*I*)
*R*_int_ = 0.055

#### Refinement

*R*[*F* ^2^ > 2σ(*F* ^2^)] = 0.043
*wR*(*F* ^2^) = 0.093
*S* = 1.03
13546 reflections
547 parameters
H atoms treated by a mixture of independent and constrained refinement
Δρ_max_ = 0.60 e Å^−3^
Δρ_min_ = −0.77 e Å^−3^

### 

Data collection: *APEX2* (Bruker, 2005 ▶); cell refinement: *SAINT* (Bruker, 2005 ▶); data reduction: *SAINT*; program(s) used to solve structure: *SHELXS97* (Sheldrick, 2008 ▶); program(s) used to refine structure: *SHELXL97* (Sheldrick, 2008 ▶); molecular graphics: *SHELXTL* (Sheldrick, 2008 ▶); software used to prepare material for publication: *SHELXTL* and *PLATON* (Spek, 2009 ▶).

## Supplementary Material

Crystal structure: contains datablock(s) I, global. DOI: 10.1107/S1600536811055395/is5023sup1.cif

Structure factors: contains datablock(s) I. DOI: 10.1107/S1600536811055395/is5023Isup2.hkl

Additional supplementary materials:  crystallographic information; 3D view; checkCIF report

## Figures and Tables

**Table 1:** Hydrogen-bond geometry (Å, °)

*D*—H⋯*A*	*D*—H	H⋯*A*	*D*⋯*A*	*D*—H⋯*A*
O2*B*—H2*OB*⋯Cl4	0.80 (3)	2.21 (3)	2.9622 (18)	158 (3)
N8*B*—H8*NB*⋯Cl1^i^	0.84 (3)	2.44 (3)	3.215 (2)	153 (2)
N8*A*—H8*NA*⋯Cl2^ii^	0.81 (3)	2.37 (3)	3.132 (2)	158 (3)
N4*B*—H4*NB*⋯Cl4^iii^	0.84 (3)	2.39 (2)	3.169 (2)	155 (2)
N4*A*—H4*NA*⋯Cl3^i^	0.83 (3)	2.46 (3)	3.217 (2)	153 (2)
O1*B*—H1*OB*⋯Cl3	0.80 (3)	2.22 (3)	3.0018 (19)	164 (3)
O2*A*—H2*OA*⋯Cl1^iv^	0.82 (3)	2.22 (3)	3.0172 (18)	165 (3)
O1*A*—H1*OA*⋯Cl2^iv^	0.78 (3)	2.19 (3)	2.9435 (18)	163 (3)
N7*A*—H7*NA*⋯Cl2^ii^	0.87 (3)	2.35 (3)	3.153 (2)	155 (2)
N3*B*—H3*NB*⋯Cl4^iii^	0.88 (3)	2.33 (3)	3.150 (2)	154 (2)
N7*B*—H7*NB*⋯Cl1^i^	0.84 (3)	2.31 (2)	3.1023 (19)	160 (2)
N3*A*—H3*NA*⋯Cl3^i^	0.85 (3)	2.27 (2)	3.0774 (19)	160 (2)
C12*A*—H12*B*⋯O2*B*^v^	0.98	2.43	3.328 (3)	153
C6*B*—H6*BB*⋯Cl2^iii^	0.98	2.70	3.598 (3)	153
C6*B*—H6*BC*⋯O1*A*	0.98	2.55	3.277 (3)	131

Symmetry codes: (i) 
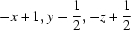
; (ii) 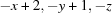; (iii) 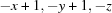; (iv) 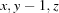; (v) 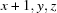.

**Fig. 1.**
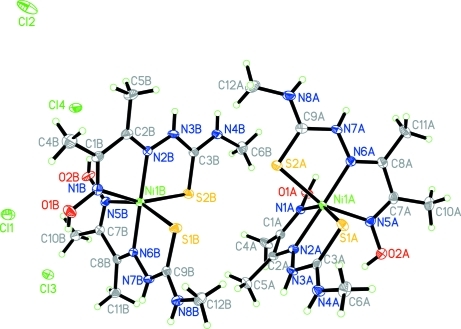
The molecular structure of the title compound, with 50% probability displacement ellipsoids and the atom-numbering scheme.

**Fig. 2.**
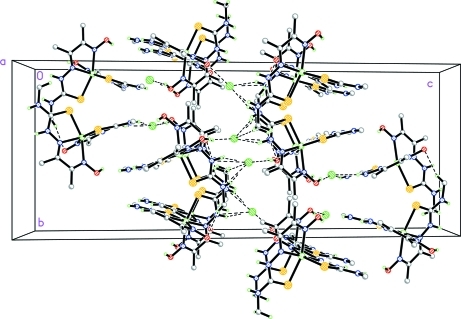
The crystal packing of the title compound viewed down the a axis. Hydrogen bonds are shown as dashed lines.

## supplementary crystallographic information

### Comment

Thiosemicarbazones and their metal complexes have attracted significant attention because of their wide-ranging biological and pharmacological properties, analytical applications, specific structures, and chemical properties (Cowley *et al.*, 2002; Ming, 2003; Lobana *et al.*, 2007; Lobana *et al.*, 2004). Recently, we reported the crystal structure of bis{*N*-ethyl-2-[2-(hydroxyimino-κ*N*)butan-2-ylidene] hydrazinecarbothioamide-κ^2^*N*^2^,*S*}nickel(II) dichloride (Abduelftah *et al.*, 2012*a*). In this paper we report the crystal structure of bis{*N*-methyl-2-[2-(hydroxyimino-κ*N*)butan-2-ylidene] hydrazinecarbothioamide-κ^2^*N*^2^,*S*}nickel(II) dichloride.

The asymmetric unit of the title compound (Fig. 1), [Ni(C_6_H_12_N_4_OS)_2_]Cl_2_, contains two Ni(II) complexes and four chloride anions. Each Ni(II) ion is six-coordinated in a distorted octahedral geometry by four N atoms from the two imine and two oxime groups and two S atoms from the thione group. The bond distances around Ni(II) ions in molecule A, B and the related Ni(II) complex (Abduelftah *et al.*, 2012*a*) as follows: Ni1—N1 [2.1226 (17), 2.1062 (18), 2.1247 (14) Å], Ni1—N2 [2.0063 (18), 2.0167 (18), 2.0120 (12) Å], Ni1—N5 [2.1185 (19), 2.1242 (17), 2.1258 (13) Å], Ni1—N6 [2.0040 (18), 2.0101 (18), 2.0086 (12) Å], Ni1—S1 [2.4191 (6), 2.4279 (6), 2.4089 (5) Å] and Ni1—S2 [2.4140 (6), 2.4129 (6), 2.4126 (5) Å]. Bond lengths and angles observed in the structure are normal (Allen *et al.*, 1987). In molecule A, the Ni(II) ion is a meeting-point of four five-membered rings, namely: A (Ni1A/S1A/N2A/N3A/C9A), B (Ni1A/S2A/N6A/N7A/C3A), C (Ni1A/N1A/N2A/C1A/C9A) and D (Ni1A/N5A/N6A/C7A/C8A). The dihedral angles between these four rings as follows: A/B = 84.64 (6)°, A/C = 6.16 (8)°, A/D = 88.00 (7)°, B/C = 89.47 (8)°, B/D = 3.48 (7)° and C/D = 86.08 (9)°. In molecule B, the Ni(II) ion is a meeting-point of four five-membered rings, namely: E (Ni1B/S1B/N6B/N7B/C9B), F (Ni1B/S2B/N2B/N3B/C3B), G (Ni1B/N1B/N2B/C1B/C2B) and H(Ni1B/N5B/N6B/C7B/C8B). The dihedral angles between these four rings as follows: E/F = 85.84 (6)°, E/G = 86.77 (7)°, E/H = 6.20 (8)°, F/G = 2.09 (7)°, F/H = 88.81 (8)° and G/H = 87.78 (9)°. In the crystal, molecules are linked through intermolecular N—H···Cl and O—H···Cl hydrogen bonds (Table 1) into infinite chains propagating in [101] (Fig. 2). C—H···O and C—H···Cl hydrogen bonds are also observed.

### Experimental

To a solution of the ligand (0.2021 g; Abduelftah *et al.*, 2012*b*) in EtOH (20 ml) was added a solution of (NiCl_2_.6H_2_O) (0.2377 g) in EtOH (20 ml). The mixture was boiled under reflux for 2 h with stirring. The mixture was filtered and left to cool accompanied by slow evaporation of the solvent at room temperature. The brown crystals were grown in DMF–acetone (1:4) mixture by slow evaporation at room temperature for 2 weeks (yield 45%; m.p. 513.9 K).

### Refinement

N- and O-bound H atoms were located in a difference Fourier map and were refined freely. H atoms of the methyl groups were positioned geometrically (C—H = 0.98 Å) and refined using a riding model, with *U*_iso_(H) = 1.5*U*_eq_(C). The highest residual electron density peak is located 1.11 Å from Ni1A and the deepest hole is located 0.70 Å from Ni1B.

### Crystal data

**Table:**

[Ni(C_6_H_12_N_4_OS)_2_]Cl_2_	*F*(000) = 2096
*M**_r_* = 506.12	*D*_x_ = 1.543 Mg m^−^^3^
Monoclinic, *P*2_1_/*c*	Mo *K*α radiation, λ = 0.71073 Å
Hall symbol: -P 2ybc	Cell parameters from 9957 reflections
*a* = 8.9484 (1) Å	θ = 2.3–30.4°
*b* = 13.8043 (2) Å	µ = 1.35 mm^−^^1^
*c* = 35.4643 (5) Å	*T* = 100 K
β = 95.780 (1)°	Plate, brown
*V* = 4358.5 (1) Å^3^	0.43 × 0.16 × 0.06 mm
*Z* = 8	

### Data collection

**Table:**

Bruker APEXII CCD diffractometer	13546 independent reflections
Radiation source: fine-focus sealed tube	9309 reflections with *I* > 2σ(*I*)
graphite	*R*_int_ = 0.055
φ and ω scans	θ_max_ = 30.9°, θ_min_ = 1.6°
Absorption correction: multi-scan (*SADABS*; Bruker, 2005)	*h* = −12→12
*T*_min_ = 0.595, *T*_max_ = 0.925	*k* = −15→19
53587 measured reflections	*l* = −32→51

### Refinement

**Table:**

Refinement on *F*^2^	Primary atom site location: structure-invariant direct methods
Least-squares matrix: full	Secondary atom site location: difference Fourier map
*R*[*F*^2^ > 2σ(*F*^2^)] = 0.043	Hydrogen site location: inferred from neighbouring sites
*wR*(*F*^2^) = 0.093	H atoms treated by a mixture of independent and constrained refinement
*S* = 1.02	*w* = 1/[σ^2^(*F*_o_^2^) + (0.032*P*)^2^ + 1.9473*P*] where *P* = (*F*_o_^2^ + 2*F*_c_^2^)/3
13546 reflections	(Δ/σ)_max_ = 0.001
547 parameters	Δρ_max_ = 0.60 e Å^−^^3^
0 restraints	Δρ_min_ = −0.77 e Å^−^^3^

### Special details

**Table:**

Geometry. All e.s.d.'s (except the e.s.d. in the dihedral angle between two l.s. planes) are estimated using the full covariance matrix. The cell e.s.d.'s are taken into account individually in the estimation of e.s.d.'s in distances, angles and torsion angles; correlations between e.s.d.'s in cell parameters are only used when they are defined by crystal symmetry. An approximate (isotropic) treatment of cell e.s.d.'s is used for estimating e.s.d.'s involving l.s. planes.
Refinement. Refinement of *F*^2^ against ALL reflections. The weighted *R*-factor *wR* and goodness of fit *S* are based on *F*^2^, conventional *R*-factors *R* are based on *F*, with *F* set to zero for negative *F*^2^. The threshold expression of *F*^2^ > σ(*F*^2^) is used only for calculating *R*-factors(gt) *etc*. and is not relevant to the choice of reflections for refinement. *R*-factors based on *F*^2^ are statistically about twice as large as those based on *F*, and *R*- factors based on ALL data will be even larger.

### Fractional atomic coordinates and isotropic or equivalent isotropic displacement parameters (Å^2^)

**Table:**

	*x*	*y*	*z*	*U*_iso_*/*U*_eq_	
Ni1A	0.93571 (3)	0.02340 (2)	0.135210 (8)	0.01214 (7)	
S1A	1.14597 (6)	0.06246 (4)	0.180994 (16)	0.01747 (12)	
S2A	0.92335 (6)	0.18150 (4)	0.105972 (16)	0.01773 (12)	
O1A	0.65049 (18)	−0.06156 (12)	0.08341 (5)	0.0171 (3)	
O2A	0.94387 (19)	−0.19028 (12)	0.16956 (5)	0.0216 (4)	
N1A	0.71047 (19)	−0.01030 (13)	0.11489 (5)	0.0128 (4)	
N2A	0.81803 (19)	0.05963 (13)	0.17836 (5)	0.0131 (4)	
N3A	0.8887 (2)	0.09436 (13)	0.21176 (5)	0.0140 (4)	
N4A	1.1023 (2)	0.11042 (15)	0.25155 (6)	0.0182 (4)	
N5A	0.97820 (19)	−0.12704 (13)	0.14133 (5)	0.0159 (4)	
N6A	1.05400 (19)	−0.00886 (13)	0.09181 (5)	0.0141 (4)	
N7A	1.0824 (2)	0.06073 (14)	0.06613 (6)	0.0169 (4)	
N8A	1.0399 (2)	0.20880 (15)	0.04065 (6)	0.0202 (4)	
C1A	0.6122 (2)	0.01093 (16)	0.13770 (6)	0.0141 (4)	
C2A	0.6728 (2)	0.05926 (15)	0.17329 (6)	0.0137 (4)	
C3A	1.0422 (2)	0.08939 (15)	0.21673 (6)	0.0138 (4)	
C4A	0.4489 (2)	−0.01454 (19)	0.13136 (7)	0.0220 (5)	
H4AA	0.4349	−0.0680	0.1132	0.033*	
H4AB	0.3917	0.0420	0.1213	0.033*	
H4AC	0.4131	−0.0342	0.1554	0.033*	
C5A	0.5717 (2)	0.09987 (17)	0.20013 (6)	0.0193 (5)	
H5AA	0.6243	0.1512	0.2153	0.029*	
H5AB	0.5425	0.0484	0.2170	0.029*	
H5AC	0.4817	0.1267	0.1858	0.029*	
C6A	1.2634 (2)	0.11115 (18)	0.26292 (7)	0.0227 (5)	
H6AA	1.2817	0.1304	0.2896	0.034*	
H6AB	1.3124	0.1573	0.2471	0.034*	
H6AC	1.3044	0.0462	0.2597	0.034*	
C7A	1.0628 (2)	−0.16390 (17)	0.11782 (6)	0.0184 (5)	
C8A	1.1041 (2)	−0.09592 (17)	0.08818 (6)	0.0170 (5)	
C9A	1.0192 (2)	0.14997 (16)	0.06907 (6)	0.0163 (5)	
C10A	1.1189 (3)	−0.26575 (19)	0.11891 (8)	0.0317 (6)	
H10A	1.0784	−0.3006	0.1397	0.048*	
H10B	1.2289	−0.2657	0.1229	0.048*	
H10C	1.0863	−0.2978	0.0948	0.048*	
C11A	1.1935 (3)	−0.12730 (19)	0.05683 (7)	0.0255 (6)	
H11A	1.2751	−0.0811	0.0545	0.038*	
H11B	1.1280	−0.1296	0.0330	0.038*	
H11C	1.2357	−0.1918	0.0625	0.038*	
C12A	0.9922 (3)	0.30916 (18)	0.03797 (7)	0.0283 (6)	
H12A	0.9780	0.3286	0.0113	0.043*	
H12B	1.0690	0.3502	0.0516	0.043*	
H12C	0.8972	0.3165	0.0492	0.043*	
Ni1B	0.45084 (3)	0.42366 (2)	0.128901 (8)	0.01295 (7)	
S1B	0.66334 (6)	0.38150 (4)	0.173946 (16)	0.01794 (12)	
S2B	0.43668 (6)	0.26579 (4)	0.099552 (16)	0.01829 (12)	
O1B	0.4669 (2)	0.63521 (13)	0.16411 (5)	0.0226 (4)	
O2B	0.16117 (18)	0.50443 (12)	0.07736 (5)	0.0206 (4)	
N1B	0.49734 (19)	0.57262 (13)	0.13547 (5)	0.0159 (4)	
N2B	0.56170 (19)	0.45676 (13)	0.08383 (5)	0.0139 (4)	
N3B	0.5824 (2)	0.38836 (14)	0.05714 (6)	0.0173 (4)	
N4B	0.5596 (2)	0.23501 (14)	0.03515 (6)	0.0191 (4)	
N5B	0.22468 (19)	0.45904 (13)	0.10994 (5)	0.0150 (4)	
N6B	0.33650 (19)	0.38842 (13)	0.17291 (5)	0.0131 (4)	
N7B	0.4084 (2)	0.35576 (13)	0.20647 (5)	0.0142 (4)	
N8B	0.6243 (2)	0.33922 (14)	0.24541 (6)	0.0179 (4)	
C1B	0.5796 (2)	0.60989 (16)	0.11130 (6)	0.0170 (5)	
C2B	0.6153 (2)	0.54335 (17)	0.08077 (6)	0.0175 (5)	
C3B	0.5300 (2)	0.29632 (16)	0.06207 (6)	0.0160 (5)	
C4B	0.6354 (3)	0.71179 (18)	0.11254 (8)	0.0295 (6)	
H4BA	0.6047	0.7441	0.1351	0.044*	
H4BB	0.5929	0.7463	0.0898	0.044*	
H4BC	0.7453	0.7119	0.1135	0.044*	
C5B	0.7050 (3)	0.57521 (18)	0.04949 (7)	0.0238 (5)	
H5BA	0.6410	0.5746	0.0254	0.036*	
H5BB	0.7897	0.5309	0.0479	0.036*	
H5BC	0.7429	0.6410	0.0547	0.036*	
C6B	0.5288 (3)	0.13162 (17)	0.03615 (7)	0.0250 (5)	
H6BA	0.5838	0.0984	0.0174	0.038*	
H6BB	0.4209	0.1205	0.0303	0.038*	
H6BC	0.5612	0.1063	0.0615	0.038*	
C7B	0.1283 (2)	0.43598 (16)	0.13289 (6)	0.0155 (5)	
C8B	0.1917 (2)	0.38909 (15)	0.16863 (6)	0.0141 (4)	
C9B	0.5619 (2)	0.35872 (15)	0.21064 (6)	0.0138 (4)	
C10B	−0.0360 (2)	0.45858 (18)	0.12681 (7)	0.0217 (5)	
H10D	−0.0545	0.5048	0.1059	0.033*	
H10E	−0.0686	0.4869	0.1500	0.033*	
H10F	−0.0924	0.3989	0.1206	0.033*	
C11B	0.0934 (2)	0.35021 (17)	0.19646 (7)	0.0203 (5)	
H11D	0.1371	0.2904	0.2076	0.030*	
H11E	−0.0065	0.3365	0.1836	0.030*	
H11F	0.0848	0.3981	0.2165	0.030*	
C12B	0.7865 (2)	0.33501 (17)	0.25571 (7)	0.0208 (5)	
H12D	0.8067	0.3153	0.2823	0.031*	
H12E	0.8304	0.3990	0.2523	0.031*	
H12F	0.8312	0.2879	0.2395	0.031*	
Cl1	0.67158 (6)	0.85286 (4)	0.210434 (16)	0.02176 (13)	
Cl2	0.82262 (7)	0.92879 (6)	0.017017 (17)	0.03213 (16)	
Cl3	0.19959 (7)	0.59593 (4)	0.206586 (16)	0.02273 (13)	
Cl4	0.33146 (6)	0.62294 (5)	0.026723 (16)	0.02414 (13)	
H2OB	0.226 (3)	0.527 (2)	0.0662 (8)	0.036 (9)*	
H8NB	0.567 (3)	0.3322 (19)	0.2626 (8)	0.027 (8)*	
H8NA	1.085 (3)	0.1876 (19)	0.0239 (7)	0.021 (8)*	
H4NB	0.602 (3)	0.2559 (18)	0.0166 (7)	0.022 (7)*	
H4NA	1.045 (3)	0.1157 (18)	0.2683 (7)	0.018 (7)*	
H1OB	0.399 (3)	0.613 (2)	0.1746 (9)	0.044 (10)*	
H2OA	0.875 (3)	−0.168 (2)	0.1805 (9)	0.044 (10)*	
H1OA	0.711 (3)	−0.060 (2)	0.0689 (9)	0.043 (10)*	
H7NA	1.109 (3)	0.0436 (19)	0.0442 (8)	0.028 (8)*	
H3NB	0.618 (3)	0.4044 (19)	0.0357 (8)	0.029 (8)*	
H7NB	0.364 (3)	0.3531 (17)	0.2261 (7)	0.015 (7)*	
H3NA	0.842 (3)	0.0964 (17)	0.2313 (7)	0.015 (7)*	

### Atomic displacement parameters (Å^2^)

**Table:**

	*U*^11^	*U*^22^	*U*^33^	*U*^12^	*U*^13^	*U*^23^
Ni1A	0.01211 (12)	0.01390 (15)	0.01070 (14)	0.00079 (11)	0.00253 (10)	−0.00175 (11)
S1A	0.0123 (2)	0.0244 (3)	0.0158 (3)	0.0009 (2)	0.0017 (2)	−0.0053 (2)
S2A	0.0214 (3)	0.0159 (3)	0.0170 (3)	0.0009 (2)	0.0071 (2)	0.0001 (2)
O1A	0.0192 (8)	0.0195 (9)	0.0128 (8)	−0.0046 (7)	0.0022 (7)	−0.0046 (7)
O2A	0.0249 (9)	0.0197 (9)	0.0205 (9)	0.0041 (7)	0.0045 (8)	0.0033 (7)
N1A	0.0157 (8)	0.0125 (9)	0.0101 (9)	0.0003 (7)	0.0012 (7)	−0.0013 (7)
N2A	0.0144 (8)	0.0143 (9)	0.0106 (9)	−0.0019 (7)	0.0012 (7)	−0.0007 (7)
N3A	0.0148 (8)	0.0188 (10)	0.0087 (9)	−0.0001 (7)	0.0031 (7)	−0.0025 (8)
N4A	0.0184 (9)	0.0231 (11)	0.0127 (10)	0.0005 (8)	0.0007 (8)	−0.0031 (8)
N5A	0.0173 (8)	0.0168 (10)	0.0130 (9)	0.0009 (7)	−0.0013 (7)	0.0012 (8)
N6A	0.0142 (8)	0.0160 (10)	0.0121 (9)	−0.0002 (7)	0.0006 (7)	−0.0013 (8)
N7A	0.0209 (9)	0.0197 (11)	0.0110 (9)	0.0019 (8)	0.0060 (8)	0.0001 (8)
N8A	0.0259 (10)	0.0225 (11)	0.0128 (10)	−0.0009 (9)	0.0058 (9)	−0.0003 (9)
C1A	0.0129 (9)	0.0142 (11)	0.0152 (11)	0.0001 (8)	0.0023 (8)	0.0013 (9)
C2A	0.0159 (9)	0.0137 (11)	0.0123 (10)	−0.0004 (8)	0.0042 (8)	0.0009 (9)
C3A	0.0170 (10)	0.0098 (10)	0.0144 (11)	0.0014 (8)	0.0008 (8)	0.0004 (9)
C4A	0.0133 (10)	0.0343 (15)	0.0190 (12)	−0.0052 (10)	0.0048 (9)	−0.0074 (11)
C5A	0.0185 (10)	0.0195 (12)	0.0206 (12)	0.0012 (9)	0.0058 (9)	−0.0041 (10)
C6A	0.0199 (11)	0.0226 (13)	0.0235 (13)	0.0002 (10)	−0.0077 (10)	−0.0021 (10)
C7A	0.0192 (10)	0.0180 (12)	0.0171 (11)	0.0049 (9)	−0.0032 (9)	−0.0035 (9)
C8A	0.0152 (10)	0.0220 (12)	0.0133 (11)	0.0028 (9)	−0.0011 (8)	−0.0040 (9)
C9A	0.0155 (10)	0.0193 (12)	0.0138 (11)	−0.0046 (9)	0.0006 (9)	−0.0008 (9)
C10A	0.0403 (15)	0.0244 (14)	0.0312 (15)	0.0159 (12)	0.0070 (12)	0.0010 (12)
C11A	0.0225 (11)	0.0346 (15)	0.0199 (13)	0.0077 (11)	0.0052 (10)	−0.0083 (11)
C12A	0.0398 (14)	0.0239 (14)	0.0214 (13)	−0.0010 (11)	0.0034 (12)	0.0060 (11)
Ni1B	0.01284 (12)	0.01463 (15)	0.01155 (14)	−0.00063 (11)	0.00200 (10)	0.00137 (11)
S1B	0.0129 (2)	0.0254 (3)	0.0157 (3)	0.0001 (2)	0.0021 (2)	0.0053 (2)
S2B	0.0224 (3)	0.0158 (3)	0.0177 (3)	−0.0018 (2)	0.0073 (2)	0.0004 (2)
O1B	0.0266 (9)	0.0219 (10)	0.0200 (9)	−0.0024 (7)	0.0058 (8)	−0.0070 (7)
O2B	0.0184 (8)	0.0239 (9)	0.0187 (9)	−0.0032 (7)	−0.0016 (7)	0.0104 (7)
N1B	0.0159 (8)	0.0156 (10)	0.0156 (9)	−0.0009 (7)	−0.0010 (7)	−0.0021 (8)
N2B	0.0146 (8)	0.0131 (9)	0.0142 (9)	0.0009 (7)	0.0021 (7)	0.0015 (7)
N3B	0.0246 (10)	0.0159 (10)	0.0127 (10)	−0.0022 (8)	0.0081 (8)	0.0001 (8)
N4B	0.0268 (10)	0.0179 (11)	0.0135 (10)	0.0018 (8)	0.0058 (9)	−0.0008 (8)
N5B	0.0181 (9)	0.0132 (9)	0.0132 (9)	0.0006 (7)	−0.0002 (7)	0.0018 (8)
N6B	0.0152 (8)	0.0134 (9)	0.0107 (9)	0.0017 (7)	0.0015 (7)	0.0000 (7)
N7B	0.0137 (8)	0.0188 (10)	0.0103 (9)	0.0007 (7)	0.0021 (7)	0.0031 (8)
N8B	0.0189 (9)	0.0204 (11)	0.0142 (10)	−0.0001 (8)	0.0008 (8)	0.0021 (8)
C1B	0.0177 (10)	0.0162 (12)	0.0167 (11)	−0.0043 (9)	−0.0003 (9)	0.0022 (9)
C2B	0.0157 (10)	0.0197 (12)	0.0172 (11)	−0.0010 (9)	0.0016 (9)	0.0034 (9)
C3B	0.0169 (10)	0.0178 (12)	0.0133 (11)	0.0021 (9)	0.0013 (9)	0.0019 (9)
C4B	0.0359 (14)	0.0200 (13)	0.0342 (15)	−0.0107 (11)	0.0108 (12)	−0.0031 (12)
C5B	0.0264 (12)	0.0223 (13)	0.0235 (13)	−0.0056 (10)	0.0065 (10)	0.0060 (11)
C6B	0.0334 (13)	0.0154 (12)	0.0264 (14)	0.0000 (10)	0.0034 (11)	−0.0046 (10)
C7B	0.0157 (10)	0.0152 (11)	0.0156 (11)	0.0002 (8)	0.0013 (9)	0.0005 (9)
C8B	0.0145 (9)	0.0124 (11)	0.0157 (11)	0.0012 (8)	0.0033 (8)	−0.0009 (9)
C9B	0.0144 (9)	0.0111 (11)	0.0162 (11)	−0.0009 (8)	0.0019 (8)	−0.0011 (9)
C10B	0.0147 (10)	0.0289 (14)	0.0217 (12)	0.0017 (9)	0.0022 (9)	0.0073 (11)
C11B	0.0176 (10)	0.0230 (13)	0.0211 (12)	0.0006 (9)	0.0062 (9)	0.0058 (10)
C12B	0.0186 (10)	0.0225 (13)	0.0201 (12)	0.0004 (9)	−0.0040 (9)	0.0031 (10)
Cl1	0.0310 (3)	0.0195 (3)	0.0160 (3)	−0.0022 (2)	0.0084 (2)	0.0006 (2)
Cl2	0.0221 (3)	0.0597 (5)	0.0152 (3)	−0.0021 (3)	0.0054 (2)	−0.0100 (3)
Cl3	0.0329 (3)	0.0210 (3)	0.0158 (3)	0.0038 (2)	0.0094 (2)	0.0003 (2)
Cl4	0.0276 (3)	0.0287 (3)	0.0171 (3)	0.0043 (2)	0.0068 (2)	0.0075 (2)

### Geometric parameters (Å, °)

**Table:**

Ni1A—N6A	2.0040 (18)	Ni1B—N6B	2.0101 (18)
Ni1A—N2A	2.0063 (18)	Ni1B—N2B	2.0167 (18)
Ni1A—N5A	2.1185 (19)	Ni1B—N1B	2.1062 (18)
Ni1A—N1A	2.1226 (17)	Ni1B—N5B	2.1242 (17)
Ni1A—S2A	2.4140 (6)	Ni1B—S2B	2.4129 (6)
Ni1A—S1A	2.4191 (6)	Ni1B—S1B	2.4279 (6)
S1A—C3A	1.686 (2)	S1B—C9B	1.689 (2)
S2A—C9A	1.692 (2)	S2B—C3B	1.693 (2)
O1A—N1A	1.384 (2)	O1B—N1B	1.381 (2)
O1A—H1OA	0.78 (3)	O1B—H1OB	0.80 (3)
O2A—N5A	1.386 (2)	O2B—N5B	1.385 (2)
O2A—H2OA	0.82 (3)	O2B—H2OB	0.80 (3)
N1A—C1A	1.287 (3)	N1B—C1B	1.292 (3)
N2A—C2A	1.294 (3)	N2B—C2B	1.296 (3)
N2A—N3A	1.372 (2)	N2B—N3B	1.363 (3)
N3A—C3A	1.368 (3)	N3B—C3B	1.372 (3)
N3A—H3NA	0.85 (2)	N3B—H3NB	0.88 (3)
N4A—C3A	1.329 (3)	N4B—C3B	1.322 (3)
N4A—C6A	1.457 (3)	N4B—C6B	1.455 (3)
N4A—H4NA	0.82 (2)	N4B—H4NB	0.84 (3)
N5A—C7A	1.286 (3)	N5B—C7B	1.284 (3)
N6A—C8A	1.293 (3)	N6B—C8B	1.290 (2)
N6A—N7A	1.365 (3)	N6B—N7B	1.371 (2)
N7A—C9A	1.364 (3)	N7B—C9B	1.367 (3)
N7A—H7NA	0.87 (3)	N7B—H7NB	0.84 (2)
N8A—C9A	1.322 (3)	N8B—C9B	1.329 (3)
N8A—C12A	1.450 (3)	N8B—C12B	1.462 (3)
N8A—H8NA	0.80 (3)	N8B—H8NB	0.84 (3)
C1A—C2A	1.481 (3)	C1B—C2B	1.479 (3)
C1A—C4A	1.498 (3)	C1B—C4B	1.492 (3)
C2A—C5A	1.487 (3)	C2B—C5B	1.500 (3)
C4A—H4AA	0.9800	C4B—H4BA	0.9800
C4A—H4AB	0.9800	C4B—H4BB	0.9800
C4A—H4AC	0.9800	C4B—H4BC	0.9800
C5A—H5AA	0.9800	C5B—H5BA	0.9800
C5A—H5AB	0.9800	C5B—H5BB	0.9800
C5A—H5AC	0.9800	C5B—H5BC	0.9800
C6A—H6AA	0.9800	C6B—H6BA	0.9800
C6A—H6AB	0.9800	C6B—H6BB	0.9800
C6A—H6AC	0.9800	C6B—H6BC	0.9800
C7A—C8A	1.483 (3)	C7B—C8B	1.484 (3)
C7A—C10A	1.492 (3)	C7B—C10B	1.497 (3)
C8A—C11A	1.498 (3)	C8B—C11B	1.487 (3)
C10A—H10A	0.9800	C10B—H10D	0.9800
C10A—H10B	0.9800	C10B—H10E	0.9800
C10A—H10C	0.9800	C10B—H10F	0.9800
C11A—H11A	0.9800	C11B—H11D	0.9800
C11A—H11B	0.9800	C11B—H11E	0.9800
C11A—H11C	0.9800	C11B—H11F	0.9800
C12A—H12A	0.9800	C12B—H12D	0.9800
C12A—H12B	0.9800	C12B—H12E	0.9800
C12A—H12C	0.9800	C12B—H12F	0.9800
			
N6A—Ni1A—N2A	178.40 (7)	N6B—Ni1B—N2B	178.45 (7)
N6A—Ni1A—N5A	75.97 (7)	N6B—Ni1B—N1B	105.11 (7)
N2A—Ni1A—N5A	105.60 (7)	N2B—Ni1B—N1B	76.05 (7)
N6A—Ni1A—N1A	104.33 (7)	N6B—Ni1B—N5B	76.01 (7)
N2A—Ni1A—N1A	76.12 (7)	N2B—Ni1B—N5B	103.07 (7)
N5A—Ni1A—N1A	88.53 (7)	N1B—Ni1B—N5B	88.96 (7)
N6A—Ni1A—S2A	82.87 (5)	N6B—Ni1B—S2B	96.18 (5)
N2A—Ni1A—S2A	95.59 (5)	N2B—Ni1B—S2B	82.61 (5)
N5A—Ni1A—S2A	158.47 (5)	N1B—Ni1B—S2B	158.54 (6)
N1A—Ni1A—S2A	92.95 (5)	N5B—Ni1B—S2B	93.68 (5)
N6A—Ni1A—S1A	97.46 (5)	N6B—Ni1B—S1B	81.80 (5)
N2A—Ni1A—S1A	82.24 (5)	N2B—Ni1B—S1B	99.24 (5)
N5A—Ni1A—S1A	91.74 (5)	N1B—Ni1B—S1B	91.62 (5)
N1A—Ni1A—S1A	157.59 (5)	N5B—Ni1B—S1B	157.14 (5)
S2A—Ni1A—S1A	94.92 (2)	S2B—Ni1B—S1B	94.07 (2)
C3A—S1A—Ni1A	95.94 (7)	C9B—S1B—Ni1B	96.06 (7)
C9A—S2A—Ni1A	95.44 (8)	C3B—S2B—Ni1B	96.04 (8)
N1A—O1A—H1OA	106 (2)	N1B—O1B—H1OB	109 (2)
N5A—O2A—H2OA	110 (2)	N5B—O2B—H2OB	110 (2)
C1A—N1A—O1A	112.92 (17)	C1B—N1B—O1B	113.99 (18)
C1A—N1A—Ni1A	114.98 (13)	C1B—N1B—Ni1B	115.78 (15)
O1A—N1A—Ni1A	131.60 (13)	O1B—N1B—Ni1B	129.84 (14)
C2A—N2A—N3A	119.38 (18)	C2B—N2B—N3B	120.23 (19)
C2A—N2A—Ni1A	119.35 (14)	C2B—N2B—Ni1B	119.39 (16)
N3A—N2A—Ni1A	120.92 (13)	N3B—N2B—Ni1B	120.37 (14)
C3A—N3A—N2A	117.59 (18)	N2B—N3B—C3B	118.77 (19)
C3A—N3A—H3NA	117.9 (15)	N2B—N3B—H3NB	120.8 (17)
N2A—N3A—H3NA	119.8 (16)	C3B—N3B—H3NB	120.0 (17)
C3A—N4A—C6A	123.8 (2)	C3B—N4B—C6B	123.9 (2)
C3A—N4A—H4NA	117.7 (17)	C3B—N4B—H4NB	118.9 (18)
C6A—N4A—H4NA	118.0 (17)	C6B—N4B—H4NB	117.2 (18)
C7A—N5A—O2A	114.01 (19)	C7B—N5B—O2B	113.29 (17)
C7A—N5A—Ni1A	115.64 (16)	C7B—N5B—Ni1B	114.95 (14)
O2A—N5A—Ni1A	129.86 (14)	O2B—N5B—Ni1B	131.75 (14)
C8A—N6A—N7A	119.86 (19)	C8B—N6B—N7B	118.85 (19)
C8A—N6A—Ni1A	119.86 (16)	C8B—N6B—Ni1B	119.42 (14)
N7A—N6A—Ni1A	120.29 (14)	N7B—N6B—Ni1B	121.50 (13)
C9A—N7A—N6A	118.54 (19)	C9B—N7B—N6B	117.55 (19)
C9A—N7A—H7NA	117.8 (17)	C9B—N7B—H7NB	117.8 (16)
N6A—N7A—H7NA	119.5 (17)	N6B—N7B—H7NB	121.2 (16)
C9A—N8A—C12A	125.0 (2)	C9B—N8B—C12B	123.6 (2)
C9A—N8A—H8NA	117.2 (19)	C9B—N8B—H8NB	117.8 (18)
C12A—N8A—H8NA	117.7 (19)	C12B—N8B—H8NB	118.5 (18)
N1A—C1A—C2A	115.04 (18)	N1B—C1B—C2B	114.7 (2)
N1A—C1A—C4A	124.76 (19)	N1B—C1B—C4B	124.6 (2)
C2A—C1A—C4A	120.10 (19)	C2B—C1B—C4B	120.7 (2)
N2A—C2A—C1A	113.36 (19)	N2B—C2B—C1B	113.7 (2)
N2A—C2A—C5A	125.23 (19)	N2B—C2B—C5B	124.2 (2)
C1A—C2A—C5A	121.38 (18)	C1B—C2B—C5B	122.0 (2)
N4A—C3A—N3A	114.6 (2)	N4B—C3B—N3B	113.9 (2)
N4A—C3A—S1A	122.98 (17)	N4B—C3B—S2B	123.93 (18)
N3A—C3A—S1A	122.39 (16)	N3B—C3B—S2B	122.19 (17)
C1A—C4A—H4AA	109.5	C1B—C4B—H4BA	109.5
C1A—C4A—H4AB	109.5	C1B—C4B—H4BB	109.5
H4AA—C4A—H4AB	109.5	H4BA—C4B—H4BB	109.5
C1A—C4A—H4AC	109.5	C1B—C4B—H4BC	109.5
H4AA—C4A—H4AC	109.5	H4BA—C4B—H4BC	109.5
H4AB—C4A—H4AC	109.5	H4BB—C4B—H4BC	109.5
C2A—C5A—H5AA	109.5	C2B—C5B—H5BA	109.5
C2A—C5A—H5AB	109.5	C2B—C5B—H5BB	109.5
H5AA—C5A—H5AB	109.5	H5BA—C5B—H5BB	109.5
C2A—C5A—H5AC	109.5	C2B—C5B—H5BC	109.5
H5AA—C5A—H5AC	109.5	H5BA—C5B—H5BC	109.5
H5AB—C5A—H5AC	109.5	H5BB—C5B—H5BC	109.5
N4A—C6A—H6AA	109.5	N4B—C6B—H6BA	109.5
N4A—C6A—H6AB	109.5	N4B—C6B—H6BB	109.5
H6AA—C6A—H6AB	109.5	H6BA—C6B—H6BB	109.5
N4A—C6A—H6AC	109.5	N4B—C6B—H6BC	109.5
H6AA—C6A—H6AC	109.5	H6BA—C6B—H6BC	109.5
H6AB—C6A—H6AC	109.5	H6BB—C6B—H6BC	109.5
N5A—C7A—C8A	114.4 (2)	N5B—C7B—C8B	115.20 (18)
N5A—C7A—C10A	125.1 (2)	N5B—C7B—C10B	124.72 (19)
C8A—C7A—C10A	120.5 (2)	C8B—C7B—C10B	120.0 (2)
N6A—C8A—C7A	113.8 (2)	N6B—C8B—C7B	113.37 (19)
N6A—C8A—C11A	124.0 (2)	N6B—C8B—C11B	125.03 (19)
C7A—C8A—C11A	122.2 (2)	C7B—C8B—C11B	121.58 (18)
N8A—C9A—N7A	113.9 (2)	N8B—C9B—N7B	114.7 (2)
N8A—C9A—S2A	123.54 (19)	N8B—C9B—S1B	122.99 (17)
N7A—C9A—S2A	122.55 (17)	N7B—C9B—S1B	122.28 (16)
C7A—C10A—H10A	109.5	C7B—C10B—H10D	109.5
C7A—C10A—H10B	109.5	C7B—C10B—H10E	109.5
H10A—C10A—H10B	109.5	H10D—C10B—H10E	109.5
C7A—C10A—H10C	109.5	C7B—C10B—H10F	109.5
H10A—C10A—H10C	109.5	H10D—C10B—H10F	109.5
H10B—C10A—H10C	109.5	H10E—C10B—H10F	109.5
C8A—C11A—H11A	109.5	C8B—C11B—H11D	109.5
C8A—C11A—H11B	109.5	C8B—C11B—H11E	109.5
H11A—C11A—H11B	109.5	H11D—C11B—H11E	109.5
C8A—C11A—H11C	109.5	C8B—C11B—H11F	109.5
H11A—C11A—H11C	109.5	H11D—C11B—H11F	109.5
H11B—C11A—H11C	109.5	H11E—C11B—H11F	109.5
N8A—C12A—H12A	109.5	N8B—C12B—H12D	109.5
N8A—C12A—H12B	109.5	N8B—C12B—H12E	109.5
H12A—C12A—H12B	109.5	H12D—C12B—H12E	109.5
N8A—C12A—H12C	109.5	N8B—C12B—H12F	109.5
H12A—C12A—H12C	109.5	H12D—C12B—H12F	109.5
H12B—C12A—H12C	109.5	H12E—C12B—H12F	109.5
			
N6A—Ni1A—S1A—C3A	179.79 (9)	N6B—Ni1B—S1B—C9B	−2.90 (9)
N2A—Ni1A—S1A—C3A	1.37 (9)	N2B—Ni1B—S1B—C9B	178.27 (9)
N5A—Ni1A—S1A—C3A	−104.13 (9)	N1B—Ni1B—S1B—C9B	102.14 (9)
N1A—Ni1A—S1A—C3A	−13.77 (16)	N5B—Ni1B—S1B—C9B	10.96 (16)
S2A—Ni1A—S1A—C3A	96.36 (8)	S2B—Ni1B—S1B—C9B	−98.57 (8)
N6A—Ni1A—S2A—C9A	1.65 (8)	N6B—Ni1B—S2B—C3B	178.92 (8)
N2A—Ni1A—S2A—C9A	−178.76 (8)	N2B—Ni1B—S2B—C3B	−0.10 (8)
N5A—Ni1A—S2A—C9A	−8.95 (15)	N1B—Ni1B—S2B—C3B	6.08 (15)
N1A—Ni1A—S2A—C9A	−102.43 (8)	N5B—Ni1B—S2B—C3B	102.62 (9)
S1A—Ni1A—S2A—C9A	98.57 (7)	S1B—Ni1B—S2B—C3B	−98.90 (7)
N6A—Ni1A—N1A—C1A	−175.78 (15)	N6B—Ni1B—N1B—C1B	175.68 (15)
N2A—Ni1A—N1A—C1A	2.64 (15)	N2B—Ni1B—N1B—C1B	−5.38 (15)
N5A—Ni1A—N1A—C1A	109.10 (16)	N5B—Ni1B—N1B—C1B	−109.12 (15)
S2A—Ni1A—N1A—C1A	−92.39 (15)	S2B—Ni1B—N1B—C1B	−11.7 (2)
S1A—Ni1A—N1A—C1A	18.1 (2)	S1B—Ni1B—N1B—C1B	93.74 (15)
N6A—Ni1A—N1A—O1A	13.10 (19)	N6B—Ni1B—N1B—O1B	3.42 (17)
N2A—Ni1A—N1A—O1A	−168.48 (19)	N2B—Ni1B—N1B—O1B	−177.64 (17)
N5A—Ni1A—N1A—O1A	−62.02 (18)	N5B—Ni1B—N1B—O1B	78.62 (16)
S2A—Ni1A—N1A—O1A	96.49 (17)	S2B—Ni1B—N1B—O1B	176.05 (10)
S1A—Ni1A—N1A—O1A	−153.03 (12)	S1B—Ni1B—N1B—O1B	−78.52 (16)
N5A—Ni1A—N2A—C2A	−93.13 (17)	N1B—Ni1B—N2B—C2B	4.35 (15)
N1A—Ni1A—N2A—C2A	−8.66 (16)	N5B—Ni1B—N2B—C2B	89.95 (16)
S2A—Ni1A—N2A—C2A	83.00 (17)	S2B—Ni1B—N2B—C2B	−177.97 (16)
S1A—Ni1A—N2A—C2A	177.22 (17)	S1B—Ni1B—N2B—C2B	−85.03 (15)
N5A—Ni1A—N2A—N3A	93.72 (16)	N1B—Ni1B—N2B—N3B	−176.73 (16)
N1A—Ni1A—N2A—N3A	178.19 (17)	N5B—Ni1B—N2B—N3B	−91.13 (15)
S2A—Ni1A—N2A—N3A	−90.14 (15)	S2B—Ni1B—N2B—N3B	0.95 (14)
S1A—Ni1A—N2A—N3A	4.08 (14)	S1B—Ni1B—N2B—N3B	93.89 (14)
C2A—N2A—N3A—C3A	177.05 (19)	C2B—N2B—N3B—C3B	177.19 (19)
Ni1A—N2A—N3A—C3A	−9.8 (3)	Ni1B—N2B—N3B—C3B	−1.7 (2)
N6A—Ni1A—N5A—C7A	4.63 (15)	N6B—Ni1B—N5B—C7B	−4.63 (16)
N2A—Ni1A—N5A—C7A	−175.05 (14)	N2B—Ni1B—N5B—C7B	174.09 (16)
N1A—Ni1A—N5A—C7A	109.80 (15)	N1B—Ni1B—N5B—C7B	−110.49 (16)
S2A—Ni1A—N5A—C7A	15.5 (2)	S2B—Ni1B—N5B—C7B	90.83 (16)
S1A—Ni1A—N5A—C7A	−92.62 (15)	S1B—Ni1B—N5B—C7B	−18.8 (3)
N6A—Ni1A—N5A—O2A	175.98 (17)	N6B—Ni1B—N5B—O2B	174.03 (19)
N2A—Ni1A—N5A—O2A	−3.70 (17)	N2B—Ni1B—N5B—O2B	−7.25 (19)
N1A—Ni1A—N5A—O2A	−78.85 (16)	N1B—Ni1B—N5B—O2B	68.17 (18)
S2A—Ni1A—N5A—O2A	−173.18 (10)	S2B—Ni1B—N5B—O2B	−90.51 (17)
S1A—Ni1A—N5A—O2A	78.73 (16)	S1B—Ni1B—N5B—O2B	159.88 (12)
N5A—Ni1A—N6A—C8A	−2.90 (15)	N1B—Ni1B—N6B—C8B	94.30 (17)
N1A—Ni1A—N6A—C8A	−87.68 (16)	N5B—Ni1B—N6B—C8B	9.27 (16)
S2A—Ni1A—N6A—C8A	−178.91 (15)	S2B—Ni1B—N6B—C8B	−82.99 (17)
S1A—Ni1A—N6A—C8A	87.03 (15)	S1B—Ni1B—N6B—C8B	−176.23 (17)
N5A—Ni1A—N6A—N7A	177.33 (15)	N1B—Ni1B—N6B—N7B	−91.29 (16)
N1A—Ni1A—N6A—N7A	92.56 (15)	N5B—Ni1B—N6B—N7B	−176.32 (17)
S2A—Ni1A—N6A—N7A	1.32 (13)	S2B—Ni1B—N6B—N7B	91.42 (15)
S1A—Ni1A—N6A—N7A	−92.73 (14)	S1B—Ni1B—N6B—N7B	−1.82 (15)
C8A—N6A—N7A—C9A	175.32 (18)	C8B—N6B—N7B—C9B	−178.0 (2)
Ni1A—N6A—N7A—C9A	−4.9 (2)	Ni1B—N6B—N7B—C9B	7.5 (3)
O1A—N1A—C1A—C2A	175.53 (17)	O1B—N1B—C1B—C2B	179.08 (16)
Ni1A—N1A—C1A—C2A	2.7 (2)	Ni1B—N1B—C1B—C2B	5.6 (2)
O1A—N1A—C1A—C4A	−1.0 (3)	O1B—N1B—C1B—C4B	−1.7 (3)
Ni1A—N1A—C1A—C4A	−173.82 (18)	Ni1B—N1B—C1B—C4B	−175.19 (18)
N3A—N2A—C2A—C1A	−174.24 (18)	N3B—N2B—C2B—C1B	178.26 (17)
Ni1A—N2A—C2A—C1A	12.5 (3)	Ni1B—N2B—C2B—C1B	−2.8 (2)
N3A—N2A—C2A—C5A	4.2 (3)	N3B—N2B—C2B—C5B	−1.6 (3)
Ni1A—N2A—C2A—C5A	−169.09 (17)	Ni1B—N2B—C2B—C5B	177.35 (16)
N1A—C1A—C2A—N2A	−9.7 (3)	N1B—C1B—C2B—N2B	−2.0 (3)
C4A—C1A—C2A—N2A	167.0 (2)	C4B—C1B—C2B—N2B	178.7 (2)
N1A—C1A—C2A—C5A	171.9 (2)	N1B—C1B—C2B—C5B	177.83 (19)
C4A—C1A—C2A—C5A	−11.4 (3)	C4B—C1B—C2B—C5B	−1.4 (3)
C6A—N4A—C3A—N3A	−179.0 (2)	C6B—N4B—C3B—N3B	173.59 (19)
C6A—N4A—C3A—S1A	−1.0 (3)	C6B—N4B—C3B—S2B	−6.2 (3)
N2A—N3A—C3A—N4A	−170.38 (18)	N2B—N3B—C3B—N4B	−178.15 (17)
N2A—N3A—C3A—S1A	11.6 (3)	N2B—N3B—C3B—S2B	1.6 (3)
Ni1A—S1A—C3A—N4A	175.01 (18)	Ni1B—S2B—C3B—N4B	179.02 (17)
Ni1A—S1A—C3A—N3A	−7.16 (19)	Ni1B—S2B—C3B—N3B	−0.75 (18)
O2A—N5A—C7A—C8A	−178.21 (16)	O2B—N5B—C7B—C8B	−178.69 (17)
Ni1A—N5A—C7A—C8A	−5.5 (2)	Ni1B—N5B—C7B—C8B	0.2 (2)
O2A—N5A—C7A—C10A	1.5 (3)	O2B—N5B—C7B—C10B	−2.6 (3)
Ni1A—N5A—C7A—C10A	174.28 (18)	Ni1B—N5B—C7B—C10B	176.34 (18)
N7A—N6A—C8A—C7A	−179.23 (17)	N7B—N6B—C8B—C7B	173.64 (18)
Ni1A—N6A—C8A—C7A	1.0 (2)	Ni1B—N6B—C8B—C7B	−11.8 (3)
N7A—N6A—C8A—C11A	−0.2 (3)	N7B—N6B—C8B—C11B	−4.7 (3)
Ni1A—N6A—C8A—C11A	−179.97 (16)	Ni1B—N6B—C8B—C11B	169.89 (17)
N5A—C7A—C8A—N6A	3.1 (3)	N5B—C7B—C8B—N6B	7.2 (3)
C10A—C7A—C8A—N6A	−176.7 (2)	C10B—C7B—C8B—N6B	−169.1 (2)
N5A—C7A—C8A—C11A	−175.93 (19)	N5B—C7B—C8B—C11B	−174.4 (2)
C10A—C7A—C8A—C11A	4.3 (3)	C10B—C7B—C8B—C11B	9.3 (3)
C12A—N8A—C9A—N7A	−176.1 (2)	C12B—N8B—C9B—N7B	177.7 (2)
C12A—N8A—C9A—S2A	3.8 (3)	C12B—N8B—C9B—S1B	−0.2 (3)
N6A—N7A—C9A—N8A	−173.05 (17)	N6B—N7B—C9B—N8B	170.95 (18)
N6A—N7A—C9A—S2A	7.0 (3)	N6B—N7B—C9B—S1B	−11.1 (3)
Ni1A—S2A—C9A—N8A	175.09 (17)	Ni1B—S1B—C9B—N8B	−174.03 (18)
Ni1A—S2A—C9A—N7A	−4.99 (18)	Ni1B—S1B—C9B—N7B	8.21 (19)

### Hydrogen-bond geometry (Å, °)

**Table:**

*D*—H···*A*	*D*—H	H···*A*	*D*···*A*	*D*—H···*A*
O2B—H2OB···Cl4	0.80 (3)	2.21 (3)	2.9622 (18)	158 (3)
N8B—H8NB···Cl1^i^	0.84 (3)	2.44 (3)	3.215 (2)	153 (2)
N8A—H8NA···Cl2^ii^	0.81 (3)	2.37 (3)	3.132 (2)	158 (3)
N4B—H4NB···Cl4^iii^	0.84 (3)	2.39 (2)	3.169 (2)	155 (2)
N4A—H4NA···Cl3^i^	0.83 (3)	2.46 (3)	3.217 (2)	153 (2)
O1B—H1OB···Cl3	0.80 (3)	2.22 (3)	3.0018 (19)	164 (3)
O2A—H2OA···Cl1^iv^	0.82 (3)	2.22 (3)	3.0172 (18)	165 (3)
O1A—H1OA···Cl2^iv^	0.78 (3)	2.19 (3)	2.9435 (18)	163 (3)
N7A—H7NA···Cl2^ii^	0.87 (3)	2.35 (3)	3.153 (2)	155 (2)
N3B—H3NB···Cl4^iii^	0.88 (3)	2.33 (3)	3.150 (2)	154 (2)
N7B—H7NB···Cl1^i^	0.84 (3)	2.31 (2)	3.1023 (19)	160 (2)
N3A—H3NA···Cl3^i^	0.85 (3)	2.27 (2)	3.0774 (19)	160 (2)
C12A—H12B···O2B^v^	0.98	2.43	3.328 (3)	153
C6B—H6BB···Cl2^iii^	0.98	2.70	3.598 (3)	153
C6B—H6BC···O1A	0.98	2.55	3.277 (3)	131

Symmetry codes: (i) −*x*+1, *y*−1/2, −*z*+1/2; (ii) −*x*+2, −*y*+1, −*z*; (iii) −*x*+1, −*y*+1, −*z*; (iv) *x*, *y*−1, *z*; (v) *x*+1, *y*, *z*.
